# Tumor reversion holds promise

**DOI:** 10.18632/oncotarget.131

**Published:** 2010-08-03

**Authors:** Adam Telerman, Robert Amson, Mary J.C. Hendrix

**Affiliations:** ^1^LBPA, UMR8113, Ecole Normale Supérieure, 61 Avenue du Président Wilson, 94235 Cachan, France; ^2^Children’s Memorial Research Center, Northwestern University Feinberg School of Medicine, 2300 Children’s Plaza, Box 222 Chicago, IL 60614-3394, USA

In the present issue of the journal, Makoto Noda and colleagues present a screening assay for anti-cancer drugs based on tumor reversion, identifying a series of hitherto unsuspected compounds that are of potential therapeutic interest. More specifically, the assay is based upon triggering the promoter of *Reck*, which functions as an inhibitor of metalloproteinases. Among the pharmaceutical agents that were able to activate the *Reck* promoter, 1/3 were known anti-cancer drugs which act through different cytopathic mechanisms. The second group comprises drugs that inhibit growth of bacteria, plasmodium falciparum, fungi and worms. The third category consists of “drugs related to the function of the central nervous system”.

Now, here comes the interesting and original idea of the paper. Activation of *Reck* induces “flat revertants” and Noda used this system already to clone the *K-rev* gene [1,2]. Flat revertants have a history of their own that started when investigators in the early 1960s used the NIH3T3 cells to assay the transforming potential of oncoviruses and oncogenes. A couple of years later in 1968, when Robert Pollack stepped into the field, he made a remarkable observation. Some of the cells infected with SV40 or polyoma viruses no longer showed the typical oncogenic phenotype, but instead, acquired a flat morphology [3]. These cells had also lost their oncogenic potential and were therefore named “flat revertants”. This reversion of the malignant phenotype was in line with the experiments initiated by Askanazy in teratocarcinoma cells at the beginning of the 20th century and later expanded to different species and cell types [4]. The most spectacular tumor reversion experiments have been carried out in plants by Braun [4]. Various approaches, using different biological systems [5,6,7] led to the identification of at least 300 genes that could be implicated in the tumor reversion process, including siah1, PS1, TSAP6, TCTP, Integrin receptors, and *Nodal*. Interestingly, *Nodal* is an embryonic morphogen recently found to be a key plasticity gene in aggressive tumor cells. Down-regulation of *Nodal* in this plastic phenotype results in reversion to a lineage specific cell type and tumor suppression [8].

Targeting tumor reversion genes in order to induce a suppression of the malignant phenotype has already led to the identification of a series of anti-histaminic, neuroleptic and anti-depressive drugs that inhibit intra-cellular levels of TCTP, a key gene in tumor reversion [9]. Other genes such as PS1, a predisposition gene to familial Alzheimer’s disease targeted by gamma-secretase inhibitors are tested in Kaposi sarcoma patients [4].

Tumor reversion as used here in Noda’s study [10] can be a fast-track for the identification of new anti-cancer drugs when we are still far away from understanding how reversion functions at the molecular level, and this is a major future challenge.
